# A Multipurpose Leguminous Plant for the Mediterranean Countries: *Leucaena leucocephala* as an Alternative Protein Source: A Review

**DOI:** 10.3390/ani11082230

**Published:** 2021-07-29

**Authors:** Anna De Angelis, Laura Gasco, Giuliana Parisi, Pier Paolo Danieli

**Affiliations:** 1 Dipartimento di Agricoltura, Alimentazione e Ambiente (Di3A), Università di Catania, Via Valdisavoia 5, 95123 Catania, Italy; 2 Dipartimento di Scienze Agrarie, Forestali e Alimentari (DISAFA), Università di Torino, Largo Paolo Braccini 2, 10095 Grugliasco, Italy; laura.gasco@unito.it; 3 Dipartimento di Scienze e Tecnologie Agrarie, Alimentari, Ambientali e Forestali (DAGRI), Università degli Studi di Firenze, Via delle Cascine 5, 50144 Firenze, Italy; giuliana.parisi@unifi.it; 4 Dipartimento di Scienze Agrarie e Forestali (DAFNE), Università degli Studi della Tuscia, Via S. C. de Lellis snc, 01100 Viterbo, Italy; danieli@unitus.it

**Keywords:** animal nutrition, chemical composition, leadtree, Ipil-Ipil, Mediterranean areas, SWOT analysis, fodder, mimosine, anti-nutritional factors

## Abstract

**Simple Summary:**

The need to address the shortage of protein ingredients linked to both territoriality and growing demand pushes research to focus attention on alternative protein sources, both vegetable and animal (insects). This review describes the characteristics, uses, strengths, and weaknesses of *Leucaena leucocephala*, a legume that can be used in the zootechnical field as an alternative to traditional protein sources for feed formulation.

**Abstract:**

In tropical and subtropical regions, as well as in the internal and/or marginal Mediterranean areas, one of the most important problems related to animal production is represented by the inadequate nutritional supplies. The low productivity of the animals, often connected to reduced annual growth, is, in fact, not infrequently attributable to the low nitrogen content and the high fiber content of the local plant species and crop residues that constitute the base ingredients of the rations commonly adopted by farmers. The use of the supplementation with arboreal and shrub fodder, although often containing anti-nutritional factors and toxins that limit its use, could be a profitable way to alleviate the nutritional deficiencies of the basic diets. *Leucaena leucocephala* (Lam.) De Wit is native to Central America and widely naturalized in the majority of Latin American countries. It is a legume suitable for tropical and subtropical environments including the countries of the Mediterranean area. Moreover, its spread is desirable if we consider the multiple uses to which it is suitable, the considerable amount of biomass produced, and its role in preserving the environment. The aim of this work was to highlight the characteristics of Leucaena that can justify its wide diffusion. A structured analysis of strengths and weaknesses was performed accordingly. Being a good protein source for feeding livestock, it could be a species to be introduced in the inland areas of the Mediterranean countries as an alternative protein source; the limit represented by the presence of anti-nutritional factors could be overcome by feed processing and by launching targeted research programs.

## 1. Introduction

**Figure 1:**
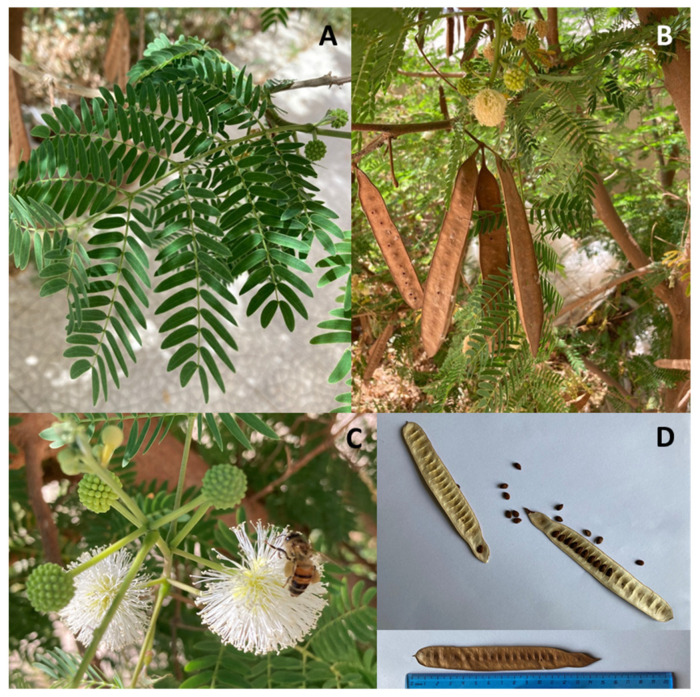
*Leucaena leucocephala* (Lam.) de Wit: (**A**) leaves; (**B**) flowers and ripened fruits; (**C**) honey bee foraging on Leucaena flowers; (**D**) pod and seeds (pictures kindly provided by Dr. Dipasquale D).

White leadtree or Leucaena (*Leucaena leucocephala*) (Figure 1), also known as ipil-ipil, is native to Central America and has spread and/or naturalized all over the World at a latitude between 30° N and 30° S due to a shrub variety used as a shade tree for coffee, cocoa, hemp plantations, pepper, vanilla, and other essences [1,2].

The *Leucaena* genus includes only 24 native species (19 diploid and five tetraploid species) [3], even though several researchers [4,5,6] refer to about 50 species of shrubs and trees found in the tropical and subtropical regions of North and South America, Africa, and the South Pacific and more than 800 cultivars, grouped in three main types: (1) the common type, with small and shrubby varieties that grow up to 5 m in height; (2) the giant type, including varieties up to 20 m tall, with larger leaves, pods, and seeds, and a larger trunk poorly branched; and (3) the “Peru” type with medium-sized varieties that grow up to 10 m in height, with lots of branches from the bottom of the trunk, and producing abundant forage with frequent pruning [1]. Undoubtedly, the morphological and ecophysiological diversities within the genus combined with the high crossability among species provide ample opportunities for genetic improvement, via traditional breeding approaches and notably via interspecific artificial hybridization, to develop genetically improved seed lines [3].

Interspecific hybridization enables plant breeders to combine superior traits from different species to form the basis of populations for the further selection and genetic improvement. Hybridization programs have been undertaken to develop new cultivars of Leucaena with the following characteristics: low mimosine forage, acid soil tolerance, psyllid resistance, cold tolerance, wood/biomass/pulp production, and sterility [7].

The optimal growth of Leucaena occurs in areas that receive an average annual precipitation of 1500 mm, with a dry season of 4 months [2] and with an average annual temperature between 25 and 30 °C [4]. It tolerates a wide variety of soil conditions [8], and the best growth occurs under direct sunlight in well-drained soils, from moderately alkaline (pH 7.5) to slightly acidic (pH 6.0), with a salinity less than 20 mmhos/cm [9].

The purpose of the review was to highlight the characteristics of Leucaena that may justify its introduction in the inland areas of the Mediterranean countries, also as an alternative to protein sources usually used in animal feed (e.g., soybean meal, alfalfa). A Strength, Weakness, Opportunity, and Threat (SWOT) analysis [10] was adopted as a structured methodology for the aforementioned purpose.

## 2. SWOT Analysis

In the following sections, pros and cons of using the Leucaena as protein source are assessed by a detailed analysis of literature data on the topic according to a four-box analysis framework approach: strengths, weakness, opportunities, and threats, known as SWOT analysis [10], as already performed by the authors elsewhere [11,12].

### 2.1. Strengths

The spread of Leucaena is mainly due to its multipurpose character. The characteristics of the wood (specific weight: 0.50−0.59 kg/cm^3^; caloric value: 19.4 kJ/g) are such to make it particularly suitable to produce coal [13,14] and paper. Being easily machinable, porous to water-soluble preservatives, and non-deformable during drying, it is used for light constructions and crates, for various types of fences, furniture, and tables [7,13]. It is also used as a shade tree in various plantations [3,7,15,16,17], to enrich the soil as a mulch [18] and nitrogen fixer [19,20,21,22], and for the reforestation of bare areas, slopes, and pastures [4,8,15]. In some rural areas of Central America and Southeast Asia, both soft seeds and leaves from Leucaena are used as vegetables in cooking; in addition, the seeds, containing more than 5.5% of fat [4,23] (Table 1), with the palmitic, stearic, behenic, lignoceric, oleic, and linoleic acids as main components, are used as a coffee substitute [19,24] and as a dewormer [25,26]. In Mexico, red, brown, and black pigments are extracted from the pods, leaves, and bark. The bark and roots are used as household medicines and the roots have emmenagogic and abortive properties [27]. Leucaena is also considered a good plant for honeybees (Figure 1).

With reference to its possible use in the agro-zootechnical field, yields in the subtropics, where temperature limitations reduce growth rates, may be only 1.5−10 t of edible fodder/ha/year [29]. Its resistance to the dry season guarantees the availability of good forage, when pasture or other forages have browned and show a reduction in their nutrient content [28]. For its high palatability, a digestibility between 50% and 70% [30,31], and its good nutritional value (22%−28% protein), Leucaena forage can be a valid feedstuff both for ruminants (cattle, sheep, and goats) [32,33] and for non-ruminants (pigs, rabbits, chickens, fish). Figueredo et al. [34] reported a change in the chemical composition of the Leucaena forage in relation to the age of the cut. In particular, between 30 and 90 days of age, they detected a significant increase in dry matter (18.78% vs. 45.47%) and a significant decrease in protein (22.90% vs. 12.31%) and ash (6.09% vs. 3.67%) but constant values of neutral detergent fiber, acid detergent fiber, hemicelluloses, cellulose, and lignin.

The high protein and β-carotene content, which make Leucaena comparable to alfalfa fodder (Table 2), is accompanied by an amino acid composition like that of soy meal and fishmeal [33], quite rich in essential amino acids such as isoleucine, leucine, phenylalanine, and histidine. Leucaena fodder can be an excellent source of calcium, phosphorus, and other minerals, depending on the mineral availability of the soil [29,30,31,34,35,36], but it is generally deficient in sodium [30,32,34]. Not much data is available on the non-structural carbohydrates present in the leaves; Kale [37] reports the following composition: 18.6% total carbohydrates, 1% starch, 2.8% total oligosaccharides, 4.2% reducing sugars, 1.2% sucrose, and 0.6% raffinose.

*L. leucocephala* is a species worthy of interest in the zootechnical field especially in tropical and subtropical regions, as well as in the internal and/or marginal ones of the Mediterranean area [38,39,40]. Table 2 and Table 3 [32,33,41,42,43,44,45,46,47,48,49] show the chemical composition of Leucaena leaves, leaf meal, forage, silage, and hay.

Dry matter productivity of Leucaena varies with soil fertility and rainfall. Edible forage yields range from 3 to 30 t DM/ha/year. In deep fertile soils receiving more than 1500 mm of evenly distributed rainfall, Leucaena produces the largest quantities of good-quality fodder. The green leaves and legumes can be used for grazing or as fodder to be distributed in the animal feeder, administered in a fresh state or in pellets. Season of the year and cutting age affect chemical composition and digestibility of Leucaena forage; Verdecia et al. [50] found that the cell wall components (neutral detergent fiber, NDF; acid detergent fiber, ADF; lignin), calcium, and silica as well as the fiber-to-nitrogen (N) ratios (NDF/N and ADF/N) increased with the regrowth age in the rainy season. Meanwhile, crude protein (CP), cell content, in vitro dry matter digestibility (IVDMD), and in situ dry matter digestibility (ISDMD) decreased; similar trends were observed for NDF, ADF, lignin, NDF/N, and ADF/N (increase with age) and for CP, cell content, and IVDMD (decrease with age) during the dry season. The maximum values for ISDMD and silica in the dry season were found at 120 days of regrowth.

For ruminants, Garcia et al. [32] found the digestible energy values of Leucaena forage ranging from 11.6 to 12.9 MJ/kg DM, values of total apparent digested crude protein (TADCP) ranging from 64.7 to 78.0%, and 42% of rumen degradable protein, with 48% of the undegradable protein being digested post-ruminally, giving a TADCP value of 70%. Possenti et al. [51] showed a reduction in methane emissions, and a consequent improvement in energy efficiency, by administering 50% of the dry substance of the diet to adult cattle of Leucaena hay, associated with ferments. McSweeney et al. [52] indicated that steers grazing on Leucaena produced approximately 28% less enteric methane than those grazing on a native grass pasture dominated by *Dicanthium* sp. In Australia, Taylor et al. [53] found a reduction of greenhouse gas emission and an increase in productivity of their herds grazing on Leucaena. In Mexico, cattle feeding with a diet based on *Pennisetum purpureum* grass supplemented with 30% foliage from Leucaena showed a decrease in methane production by 31.6% [54]. Other studies found that shifts in the bacterial populations with increases in the methanogen components are the likely basis for alterations in methanogenesis in Leucaena forage-fed cattle and identified a practical method of measuring methane emissions in grazing animals [55,56].

Heifers fed with *L. leucocephala* incorporated into the ration from 20% to 80% DM had no effect on dry matter and organic matter intake and digestibility, but energy losses in the form of methane emission were reduced to 61% (with Leucaena forage included at 80% DM level), in comparison to the no-Leucaena diet, even though the energy losses in the urine increased linearly with the increased inclusion of Leucaena in the diet [57]. The optimum level of inclusion of *L. leucocephala* in cattle rations as a source of condensed tannins lies in the range of 20–40% of ration dry matter [57].

Research carried out on buffaloes of the Murrah breed [58], fed with three levels of Leucaena leaves in their diet (0, 10, or 20 g DM/kg LBW), revealed a rapid metabolization of mimosine. Other authors [59] showed an improvement in the digestibility of the fiber and in the nitrogen balance by administering Leucaena forage added with polyethylene glycol.

In sheep, Leucaena leaves provided at a level of 20% (150 g DM/day) of the diet did not act entirely as a substitute feed, but to some extent increased the intake of basal diet itself. This would indicate that not only relatively poor degradability of Leucaena protein in the rumen makes it a valuable source of by-pass protein for supplementation of low-quality roughage, but also that the supplementation with Leucaena would improve the supply of energy to the animals [60].

Singh et al. [61] noted an increased activity of protozoa, bacteria, and rumen fungi by supplementing the diet of adult sheep with Leucaena. Santana et al. [62] found better intake and nutrient digestibility in lambs when fed with silage mixture with Leucaena and better N retention when fed alone. In lambs, the replacement of mustard seeds with Leucaena seeds, as a protein source up to 50%, did not produce adverse effects on the ingestion of the dry matter, the use of nutrients, the nitrogen balance, and the growth performance [63].

Goats are well adapted to Leucaena and can be productive on diets containing up to 100% Leucaena, because of bacterial and hepatic detoxification. Incorporation of *Leucaena* into goat production systems can improve live weight gains, milk production, worm control, and reproduction. Akingbade et al. [64] pointed out the absence of toxicosis in goat pasturing on Leucaena, justifying it with the ruminal colonization with *Synergistes jonesii*. Successful feeding systems for goats can be based on both grazed sylvo-pastoral systems and cut-and-carry intensive systems, although there is a lack of farming system research examining the integration of Leucaena into goat production systems or documentation of the feasibility of these practices [65]. In addition, other investigations on goats [66,67] observed the effects of mineral supplementation on nutritive value of Leucaena and the toxicity of 2,3-DHP. In particular, even though the addition of iodine did not increase dry matter intake, protein, and metabolizable energy digestibility, a significant and positive effect was reported as far as the nitrogen retention; also, supplementation with ferric sulphate, magnesium sulphate, and zinc sulphate appeared to prevent toxicosis, probably due to a chelating action.

In the feeding of rabbits, it is possible to include Leucaena into the diet at a rate of 25%, even if no adverse effects with inclusions of 40%−60% in balanced diets have been noted. Santos-Ricalde et al. [68] suggested that, when restricted up to 30% and supplemented with either *Moringa oleifera* or *L. leucocephala*, the growth performance remained unaffected, and the feed cost was reduced.

Raharjo et al. [69,70] attributed to Leucaena a good palatability (≥30% of the total intake) and high digestibility values when administered to rabbits. Leucaena seems to be the favorite essence of New Zealand White rabbits in the dry season [71]; to obtain better carcass characteristics, in the same breed, the level of inclusion of Leucaena must be less than 50% [72].

Al-Amin et al. [73] concluded that a pelleted diet containing 10% Leucaena leaf meal, as a replacement for soybean meals and copra cake in complete feed, improved the growth performance of New Zealand White rabbit male. As a matter of fact, the daily weight gain increased to a 10% Leucaena inclusion level and feed conversion and cost per gain decreased accordingly [73].

Using leaf meal as a supplement for broilers, a daily growth of 100−110 g per week can be determined; in addition, in quantities not exceeding 5% of the diet, leaf meal could be used with other xanthophyll sources to give a satisfactory color to the carcass [74]. Adult chickens fed with Leucaena leaf meal up to 7% of the diet showed an increase in the use of crude protein and metabolizable energy [75]. Dumorné [76] found positive effects on weight gain, body weight, and feed intake of laying hens with inclusion of Leucaena leaves meal from 6 up to 12 g/bird/day, in comparison with the control diet without Leucaena. Feeding broiler chicken with boiled *L. leucocephala* meal (10% of the diet) was recommended since it furnished high carcass and meat attributes of broiler chicken [77]. In research conducted on laying hens, a consistent increase in the yolk color, with inclusions equal to 16% of the diet [78], was found.

On growing pigs, the use of Leucaena leaves did not produce significant increases in average weight, but a 47% increase in food conversion [79]. Ekpenyong [80] claimed that it may be possible to use Leucaena leaf meal as a means of meeting the amino acid requirements of pigs fed in the tropics.

Leucaena sounds promising also for fish feeding. Isonitrogenous diets (CP about 30%) with protein from Leucaena leaf (raw and soaked) replacing from 25% to 75% animal proteins (fishmeal) were tested in Indian major carp (*Labeo rohita*) fingerlings, in a 77-day growth trial [81]. Raw Leucaena did not exhibit promising results, but soaked Leucaena leaves at the lowest inclusion level resulted better than the other diets as far as feed acceptability, growth, feed conversion, protein utilization and digestibility, and body composition, but a 50% fish meal protein replacement allowed researchers to obtain the highest economic returns due to the lower high-price fishmeal inclusion. Inoculation of bacterial strains of *Bacillus subtilis* or *B. circulans* from other fish species allowed researchers to include Leucaena leaf meal at a 30% or 40% level, replacing other ingredients in a fishmeal diet for Indian major carp with no adverse effects [82]. In Nile tilapia (*Oreochromis niloticus*) fingerlings, the replacement of berseem (*Trifolium alexandrinum*) leaf meal with Leucaena leaf meal (dried at 60 °C for 48 h or autoclaved for 15 min) resulted in a better growth rate (0.067−0.144 g/day), feed conversion ratio (1.52−2.72 g DM/g), protein efficiency (1.03−2.06), and energy utilization (9.8−18.7%) than other isonitrogenous and isoenergetic experimental diets [83]. Leucaena leaf (soaked and dried) meal was tested up to 20% inclusion in diets for African catfish fingerlings by partially replacing fishmeal, soymeal, and corn meal [84]. After 8 weeks, all the measured growth parameters, feed conversion ratio (4.33), protein efficiency ratio (0.22), and protein and fat percentages in carcass (19.15% and 12.86%, respectively) in fish resulted as better the higher the inclusion level of Leucaena leaf meal was, with no differences as far as the survival rate. Meals from Leucaena seeds sun-dried, toasted, and soaked in water or in alkaline solution were tested as ingredients for isonitrogenous diets (40% CP) in a 2-week digestibility trial using African catfish (*Clarias gariepinus*) fingerlings with no adverse effect on the specimen survival [85]. Seeds soaked in water performed better as far as the mean weight gain (0.32 g), feed conversion ratio (0.94), and energy and protein digestibility (73.6 and 70.2%, respectively), and marginal positive effects were also observed as far as the protein content of the fish carcass.

### 2.2. Weakness

**Figure 2:**
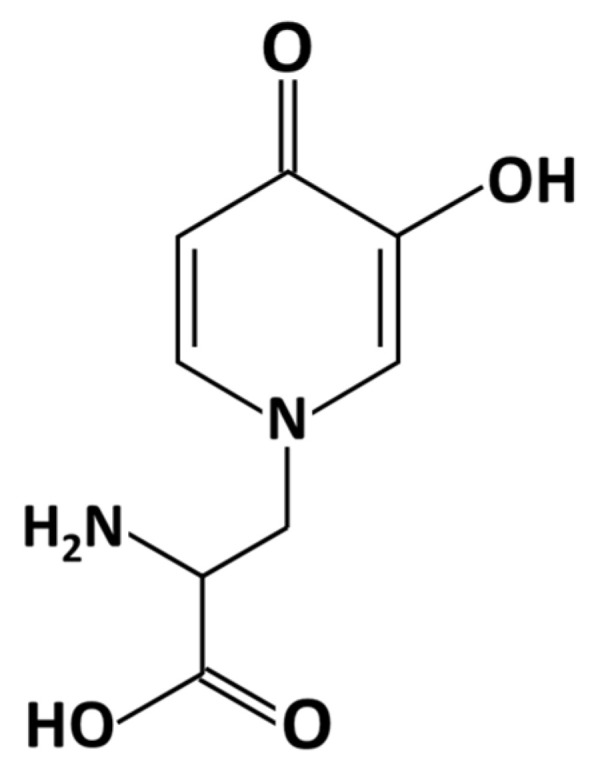
Chemical structure of β-N(3-hydroxy-4-pyridone)-α-amino propionic acid (mimosine).

The occurrence of tannins and other phenolic compounds (both in fodder and in leaf flour) can represent a limitation in the use of Leucaena for monogastric animals; in addition to tannins and phenolic compounds, the most studied and most toxic anti-nutritional factor [33] is a non-protein nitrogen compound, *i.e.*, the mimosine amino acid (Figure 2). Despite having many positive nutritional benefits, Leucaena contains the toxic non-protein free amino acid mimosine, β-N(3-hydroxy−4-pyridone)-α-amino propionic acid (Figure 2), and up to 9% dry matter (DM) in young leaves and 4−7% DM in seeds [86]. Mimosine accounts for approximately 60% of the total free amino acids (4.89 g/100 g) in *L. leucocephala* seeds [87].

Overall, ruminants fed with Leucaena showed symptoms of toxicity due to the presence of mimosine and metabolites derived from its rumen degradation: In ruminants, the primary metabolite of mimosine is the compound 3-hydroxy-4(1H)-pyridine (3,4-DHP) [88], which, in the presence of certain ruminal microbes, can be further converted to its isomer 2,3-dihydroxypyridine (2,3-DHP) [89].

Biosynthesis [90,91], degradation [92,93,94], and biochemical effects [35,95,96,97] of mimosine have been extensively examined, but to date many aspects are not yet known. The mechanism that induces toxicosis is complicated and several theories have been put forward to explain it [40].

Mimosine is acutely antimitotic [88], inhibiting the synthesis of DNA [98,99], particularly in rapidly dividing cells [100,101], and can cause damage to internal organs [102]. The symptoms ascribed to mimosine include alopecia [103,104], esophageal lesions [105], fetal abortions [106], low bull fertility [107], and death [102,105,108].

Structurally, mimosine is a tyrosine analogue [103,109], capable of inhibiting enzyme functions such as tyrosine decarboxylase and tyrosinase [110]. The inhibition of these enzymes and the inhibition of RNA synthesis in the follicle bulbs of hair cells, along with the incorporation of mimosine into biologically vital proteins [100] can result in depilation of actively growing hairs. For this reason, alopecia is one of the most reported symptoms, when animals are first introduced to Leucaena, and can occur within 7 days on 100% Leucaena-based diets [103].

The metabolite 3,4-DHP acts as a potent goitrogen. By inhibiting essential peroxidase- and lactoperoxidase-catalyzed reactions [110,111,112], the iodination of tyrosine in the binding step of the thyroid is inhibited. Compounding the goitrogenic effects of 3,4-DHP is the fact that it strongly chelates with metal ions [100], forming complexes with Zn, Cu, and Fe, in particular, leading to excretion and depletion of these minerals [113]. The 2,3-DHP has been shown to reduce dry matter intake [114] and milk production in dairy cows [115].

There are numerous studies on possible solutions to allow the use of Leucaena and to overcome its limitations due to the presence of mimosine. As an example, the heat treatment of Leucaena leaves by exposure to sunlight or high temperatures [103,116,117] can significantly reduce the content in mimosine. Wet treatments, such as cooking [118], immersion in hot water [118], and autoclave treatment [37,119], are believed to do so more effectively than dry heat treatments [35,120]. Removal and/or extraction of mimosine can be effectively accomplished with the use of 0.05 N sodium acetate [121] or urea and sodium bicarbonate [122], capable of removing high percentages of mimosine up to 80% and 88%, respectively. Silage seems to be an effective method for reducing the mimosine content in Leucaena [123]. A possible solution could also be the selective breeding of low-mimosine Leucaena hybrids [124].

In ruminants, chewing with alkaline saliva and incubation in the rumen induce the degradation of mimosine with the production of 3,4-DHP, a powerful goitrogenic [35,96,125]; nevertheless, rumen inoculations with rumen fluid of adapted animals, cultures enriched with degrading rumen bacteria, and pure cultures of *S. jonesii* have all been successfully used to create rumen populations capable of degrading 3,4-DHP and preventing Leucaena toxicosis [96,125,126,127]. Gupta and Atreja [128], working on gradual adaptation to Leucaena leaf meal in cattle, identified the presence of two types of micro-organisms degrading mimosine in 3,4-DHP and 2,3-DHP and affirmed that the type degrading 2,3-DHP can be inhibited with the presence of 3,4-DHP.

Recent studies conducted in Australia and Indonesia highlighted the possibility of avoiding the use of *S. jonesii* inoculation in cattle grazing on Leucaena, having detected a very short duration of mimosine toxicosis symptoms; they attributed this result to the contribution of other types of microorganisms in the rumen and to the conjugation process in the liver and suggested the most suitable methods for determining the presence of urinary mimosine degradation products. They suggested further research that may confirm their hypotheses [129,130,131].

### 2.3. Opportunities

Among all tropical legume plants, Leucaena probably offers the widest assortment of uses [8]. In addition, for its leafiness and copious beautiful flowers, Leucaena can be used as ornamental plant to beautify the landscape; it could be profitably widespread in the internal or marginal areas of the Mediterranean countries due to its characteristics other than to be an alternative protein source to valuable fodder for feeding polygastric and monogastric animals, being also useful for reducing methane emissions in ruminant farming, i.e.,

- It is highly productive and adaptable to various types of environmental conditions (rainfall from 250 to 1700 mm/y, neutral-alkaline soil types from rocky to heavy clay to coral) [8], with some exceptions as far as the winter cold tolerance that can limit the spread of this species at high latitudes, even though there can be some variability for the different accessions [132].
- It is useful for honeybees and other pollinating insects [133,134]. In honey from stingless bees (*Melipona* spp.) in Brazil, Leucaena pollen grains can be found at high levels (>13%) in about half of the samples [135]. In Tanzania, used in an ecological restoration program, Leucaena impacted positively on the pollinator abundance (butterflies, bees, beetles) with tangible returns in terms of Leucaena seed yield [136]. In some areas of the Yucatan peninsula, Mexico, Leucaena pollen was found to be an important protein source for the European subspecies of the honeybee (*Apis mellifera* L.) [137].
- It is a soil improver plant species [1,131] and can be planted as a living fence around the garden as ornamental, fire break, and wind break [138].
- It is useful as a dual-purpose plant, suitable for producing both biofuels and feedstuffs. Its kernel oil can be converted into biodiesel [138,139], leaving a defatted residue as a by-product that can be conveniently valorized for bioethanol production [140] or for feed-making purposes. In addition, some Leucaena cultivars, such as the Terramba, can be used for short rotation coppicing that can be conveniently integrated with the recovery of the leave mass that can be addressed to the livestock feeding [141].
- It is rich in several phytochemicals that make its seeds and leaves a promising source of pharmacological compounds also for veterinary applications. Water and hydro-alcoholic extract of Leucaena seeds exhibited good antioxidant power assessed through four different assays, partially decoupled to the tannins’ content [142]. Seed oil exhibited interesting antimicrobial activity on both mastitis caused by Gram-positive and Gram-negative bacteria such as *Staphylococcus aureus* and *Escherichia coli* [143]. Hydroalcoholic extracts of Leucaena leaves caused an average 54% reduction of the gastrointestinal nematode burden in Katahdin × Pelibuey crossbreed male lambs after 43 and 63 days of administration [144]. Protein extracts from Leucaena seeds showed anti-hatching activity on the eggs of the gastrointestinal nematode *Haemonchus contortus* in laboratory trials, probably due to the high protease and chitinase activity of the Leucaena seed extracts [145].

In such areas, as most of the research in the zootechnical field has been carried out in the countries where it is indigenous or naturalized for a long time (Mexico, Australia, Indonesia), it would be worth starting experimental programs aimed at identifying the best use.

### 2.4. Threats

Due to its high adaptability and competitivity, its invasive trait plays a role in ecosystems both under harsh [146] and wet climate and edaphic condition [147,148]. For these reasons, *L. leucocephala* is considered an invasive alien species in many countries [148,149,150]. Several studies have been conducted to identify the mechanisms that determine these traits, with the aim of finding adequate containment systems [151,152,153]. The exploitation of Leucaena for agroforestry or crop/pasture purposes should be carefully evaluated after a thorough cost-to-benefit analysis, in non-native tropical regions [148], even though it seems a minor threat in temperate and/or harsh regions wherever seasonal low temperatures or low water availability can limit the growing and diffusive potentials of this leguminous tree [146]. In temperate areas, special care should be taken to the seed propagation of Leucaena through waterways, rain washout, and unintentional cultivation [154]. The Leucaena invasive potential can be further controlled through a careful selection of more vigorous/less seed producer genotypes [155]. Due to different perspectives, to date the right exploitation of this interesting leguminous tree outside its native areal is a matter of debate [156].

## 3. Conclusions

Among the vegetable sources, *L. leucocephala* seems to be suitable to fill the deficiencies of other sources, especially from a protein and amino acid point of view, representing, moreover, an economically sustainable nutritional source. Being highly productive and having a medium-high protein content, it can be used as feedstuff, especially in those areas where the problem of finding alternative protein sources arises, such as the inland areas of the Mediterranean countries. Leucaena forage can be a valid food both for ruminants and for non-ruminants as well. Its limited use can be due to the presence of antinutritional factors, especially tannins and mimosine, but the research proved that these constraints can be overcome in several ways, not last the search of low anti-nutritional genotypes. Leucaena is also a plant worthy of interest for the reduction of methane production by ruminants when fed with Leucaena due to the presence of tannins.

Last but not least, the interest in *L. leucocephala* is linked to its versatility, which makes it a multipurpose plant that can provide several usage options: ornamental, fire break and windbreak, oil extraction and biofuel’s production, sources of pharmacological compounds also for veterinary applications, and forage plant for honeybees and other pollinators.

## Figures and Tables

**Table 1:** Chemical composition of Leucaena seeds [23,28].

Parameter	Values
Crude protein (%)	31.1 ± 0.4
Crude fat (%)	5.6 ± 0.4
Crude fiber (%)	13.2 ± 0.2
Dry matter (%)	94.8 ± 0.1
Crude ash (%)	4.5 ± 0.5
NFE (%)	40.4 ± 0.2
ME (kcal/kg)	2573.3 ± 4.2
Calcium (g/kg)	3.70 ± 0.10
Total phosphorous (g/kg)	3.400 ± 0.001
Tannin (g/kg)	7.5 ± 0.2
Phytate (mg/100 g)	697.5 ± 1.5
*Amino acids (g/kg)*	
Cystine *	3.50 ± 0.1 (1.13)
Arginine	26.2 ± 2.0 (8.42)
Methionine	3.6 ± 0.1 (1.16)
Glutamic acid	46.3 ± 0.3 (14.89)
Threonine	8.7 ± 0.1 (2.80)
Glycine	13.8 ± 0.1 (4.44)
Alanine	11.1 ± 0.1 (3.57)
Valine	11.1 ± 0.2 (3.57)
Isoleucine	9.3 ± 0.3 (2.99)
Leucine	18.1 ± 0.3 (5.82)
Lysine	13.9 ± 0.2 (4.47)
Methionine+Cystine	7.10 ± 0.02 (2.28)
*Fatty acids (% total FA)*	
C14:0	2.3
C16:0	19.6
C18:0	8.0
C18:1n-9	8.5
C18:2n-6	13.6
C18:3n-3	36.3
C20:0	2.4
SFA	41.4
MUFA	8.7
PUFA	49.9
PUFAn-6:PUFAn-3 ratio	0.4

NFE = nitrogen-free extracts; ME = metabolizable energy; SFA = saturated fatty acids; MUFA = monounsaturated fatty acids; PUFA = polyunsaturated fatty acids; * aminoacidic content in brackets is reported as g/15 gN.

**Table 2:** Chemical composition of Leucaena leaves [33,41,42,43] and leaf meal [32,44,45,46,47] in comparison with Alfalfa leaves [33].

Parameter	Leucaena Leaves	Leucaena Leaf Meal	Alfalfa Leaves
CP (%DM)	22.8–25.9	23.3–29.2	26.9
EE (%DM)	4.7	5.6–12.4	-
CF (%DM)	20.1	9.5–19.2	-
NDF (%DM)	17.4	23.6–40.4	-
ADF (%DM)	20.4–25.1	25.7–27.9	21.7
ADL (%DM)	12.8	8.3–10.5	-
ASH (%DM)	6.4–11.0	-	16.6
NFE (%DM)	46.26	40.2–48.9	-
Mimosine (%DM)	-	4.3	-
GE (MJ/kg DM)	20.1–20.2	16.2–17.8	18.5
Ca (g/kg DM)	8.0–23.6	16.0–20.8	31.5
P (g/kg DM)	2.0–3.3	2.0–2.4	3.6
Mg (g/kg DM)	1.9–4.0	3.4	-
K (g/kg DM)	-	17.0	-
Fe (mg/kg DM)	-	907.4	-
Zn (mg/kg DM)	-	19.2	-
Mn (mg/kg DM)	-	50.9–80.0	-
Phenolics (g/kg DM)	112.0	-	-
Tannins (g/kg DM)	10.2–21.0	10.1	0.13
β-carotene (mg/kg DM)	536.0	237.5	253.0
IVDMD (%)	56.8	-	-
Arginine (mg/gN)	294	-	357
Cysteine (mg/gN)	88	-	77
Histidine (mg/gN)	125	-	139
Isoleucine (mg/gN)	563	-	290
Leucine (mg/gN)	469	-	494
Lysine (mg/gN)	313	-	368
Methionine (mg/gN)	100	-	96
Phenylalanine (mg/gN)	294	-	307
Threonine (mg/gN)	231	-	290
Tyrosine (mg/gN)	263	-	232
Valine (mg/gN)	338	-	356

**Table 3:** Chemical composition (on wet weight basis, except if otherwise reported) of Leucaena forage [32], leaves’ silage [48], and hay (*L. leucocephala* cv. *Cunningham*) [49].

Parameter	Forage	Leave Silage	Hay
Dry matter (%)	-	35.22−35.65	90.55
Crude protein (%)	22.03	21.49−22.29 *	15.87
Crude fiber (%)	3.50	-	-
NDF (%)	39.50	-	48.11
ADF (%)	35.10	31.18−33.68 *	-
Hemicellulose (%)	4.71	-	-
Ether extract (%)	-	7.76−8.22 *	-
Tannins (%)	1.05	-	0.83
Mimosine (%)	2.14	-	-
Ash (%)	18.3	-	5.44
Ca (%)	1.80	-	-
P (%)	0.26	-	-
Mg (%)	0.33	-	-
K (%)	1.45	-	-
S (%)	0.22	-	-
Zn (mg/kg)	169.50	-	-
Cu (mg/kg)	26.00	-	-
Acetate (%)	-	2.00−2.90 *	-
Lactate (%)	-	6.90−9.70 *	-
Oxalates (mg/kg)	881.60	-	-

NDF = neutral detergent fiber; ADF = acid detergent fiber; * data reported on dry matter basis.

## Data Availability

Not applicable.
